# Knowing when to stop: Transcription termination on protein-coding genes by eukaryotic RNAPII

**DOI:** 10.1016/j.molcel.2022.12.021

**Published:** 2023-01-11

**Authors:** Juan B. Rodríguez-Molina, Steven West, Lori A. Passmore

**Affiliations:** 1 MRC Laboratory of Molecular Biology, Cambridge, UK; 2 The Living Systems Institute, University of Exeter, Exeter, UK

## Abstract

Gene expression is controlled in a dynamic and regulated manner to allow for the consistent and steady expression of some proteins as well as the rapidly changing production of other proteins. Transcription initiation has been a major focus of study because it is highly regulated. However, termination of transcription also plays an important role in controlling gene expression. Transcription termination on protein-coding genes is intimately linked with 3′ end cleavage and polyadenylation of transcripts, and it generally results in the production of a mature mRNA that is exported from the nucleus. Termination on many non-coding genes can also result in the production of a mature transcript. Termination is dynamically regulated—premature termination and transcription readthrough occur in response to a number of cellular signals, and these can have varied consequences on gene expression. Here, we review eukaryotic transcription termination by RNA polymerase II (RNAPII), focusing on protein-coding genes.

## Introduction

In eukaryotes, RNA polymerase II (RNAPII) is responsible for the transcription of protein-coding genes as well as many non-coding genes. It is highly regulated to allow the constitutive production of “housekeeping” genes as well as the dynamic transcription of regulatory genes in response to signals. Transcriptional control is essential in almost every cellular process, including during development, growth, and stress.

**Figure 1:**
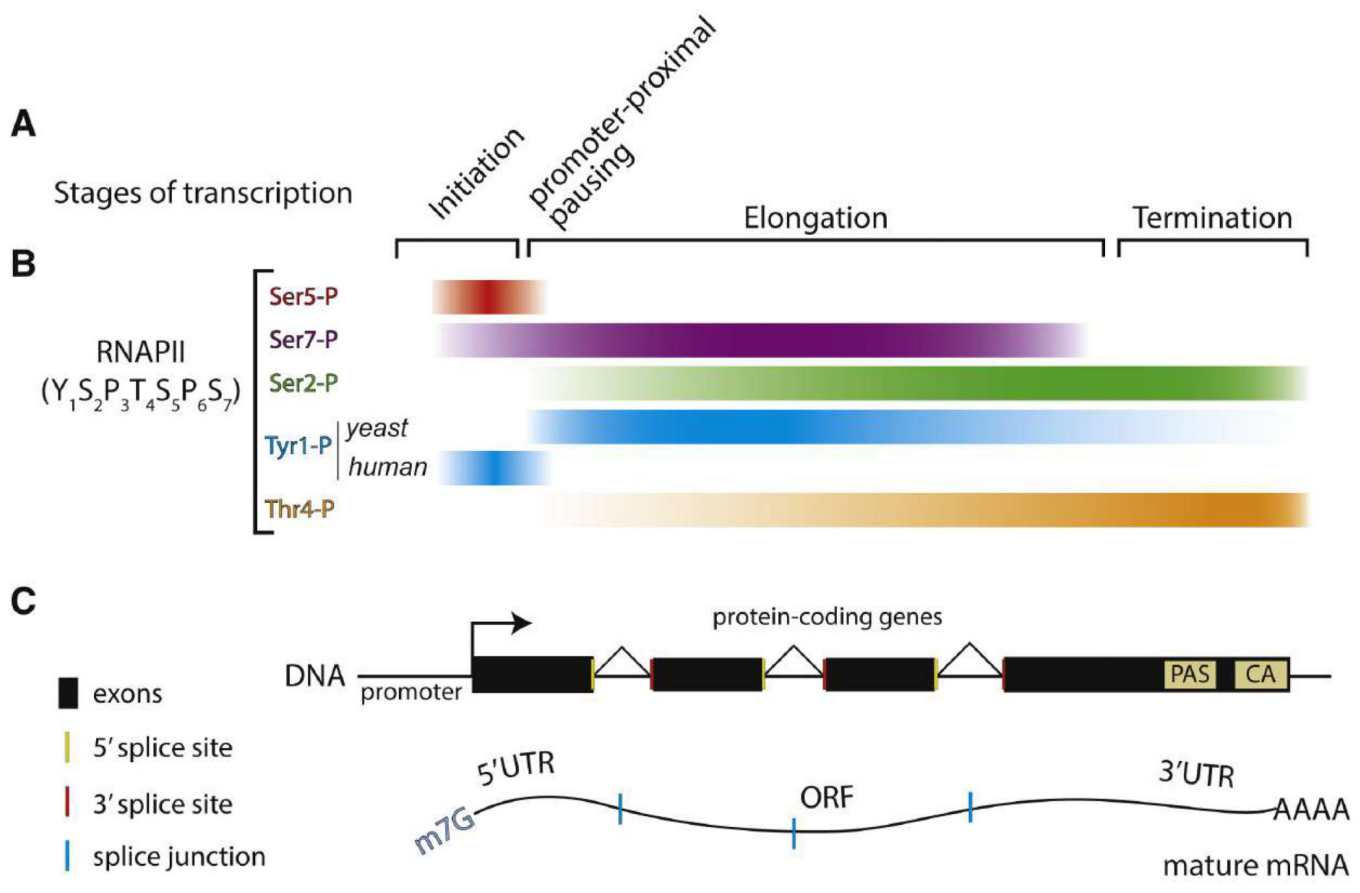
Eukaryotic transcription cycle (A) Transcription occurs in three stages, which are accompanied by the exchange of specific initiation, elongation, and termination factors. (B) The C-terminal domain (CTD) of the largest subunit of RNAPII is differentially phosphorylated throughout the transcription cycle to facilitate recruitment and exchange of stage-specific factors. Phosphorylation patterns are indicated by colored bars. (C) The coupling of transcription with phosphoCTD-mediated recruitment of mRNA processing factors enables co-transcriptional pre-mRNA processing (capping, splicing, 3′ end processing) to produce a mature mRNA. The polyadenylation signal (PAS) and the consensus sequence of the cleavage site (CA) are indicated. ORF, open reading frame; UTR, untranslated region.

Transcription can be divided into three phases: initiation, elongation, and termination (Figure 1).^1^ During transcription initiation, the preinitiation complex (PIC) assembles on promoter sequences and the promoter DNA strands are separated, allowing RNAPII to access the DNA template strand and begin RNA synthesis. RNAPII is then able to escape the promoter region, leaving many initiation factors behind.^2–4^ The transition from initiation to elongation is highly regulated: in metazoans, RNAPII pauses close to the promoter and can either be released to continue transcribing or can be removed from the gene by promoter-proximal premature termination, attenuating gene expression.^5^ After RNAPII has transcribed the remainder of the transcript, including a polyadenylation signal (PAS) sequence (most commonly AAUAAA), termination takes place.^6^

At the 3′ ends of genes, pre-mRNAs are cleaved and polyadenylated to generate a mature transcript.^7,8^ This pre-mRNA cleavage defines the end of the 3′ untranslated region (UTR) and is coupled to transcription termination. If cleavage occurs too early, it results in truncated gene products. On the other hand, if it does not occur in a timely manner, it results in transcriptional readthrough, causing interference with initiation on downstream genes or the production of extended 3′ UTRs that may contain elements that alter transcript stability. Thus, both premRNA cleavage and transcription termination must be tightly controlled.

In this review, we focus on transcription termination at the 3′ ends of protein-coding genes. We provide details on the prevailing models of transcription termination, its coupling to 3′ end processing, how it is regulated, and cases of deregulation in disease. Alternative mechanisms also facilitate termination on non-coding genes,^9^ including termination mediated by the Integrator complex (covered in an accompanying review),^5^ the ZC3H4-WDR82 restrictor complex,^10–12^ and microprocessor.^13^

## Better Together: Coupling of Transcription and Rna-Processing Events

Pre-mRNAs undergo 5′ capping, splicing, and the addition of a polyadenylate (poly(A)) tail at their 3′ end before they are exported from the nucleus as mature mRNAs. These processing events generally occur co-transcriptionally: as RNA emerges from RNAPII, it is recognized by processing factors.^14^ To enable the coupling of mRNA processing and transcription, the capping, splicing, and 3′ end processing machineries physically interact with RNAPII.^15^ Cryoelectron microscopy (cryo-EM) and biochemical studies have shown that all three of these RNA processing machineries bind next to the RNA exit tunnel of RNAPII.^16–18^ In this position, they can monitor the nascent transcript and proceed with processing as soon as the relevant sequences have been transcribed.^19^ If binding of these complexes to RNAPII is mutually exclusive, it could promote ordered and coordinated pre-mRNA processing.

The transcription cycle and co-transcriptional mRNA processing are facilitated by the C-terminal domain (CTD) of Rpb1, the largest subunit of RNAPII. The CTD is an unstructured polypeptide composed of 26- or 52-heptad repeats in yeast and human, respectively, with a consensus sequence of Tyr1-Ser2-Pro3-Thr4-Ser5-Pro6-Ser7. Differential post-translational modifications of the CTD, including phosphorylation, proline isomerization, and glycosylation, provide binding sites for transcription and pre-mRNA processing regulators throughout the transcription cycle.^20–23^ Before transcription begins, the CTD is hypophosphorylated. Soon after initiation, Ser5 and Ser7 are phosphorylated by Kin28/CDK7 within TFIIH (Figure 1B). Ser5 phosphorylation recruits the capping machinery to promote the addition of a 5′ cap and is also coincident with promoter proximal pausing in metazoans.^24,25^

At the transition from transcription initiation to elongation, the Spt5/SPT5 subunit of the DSIF elongation factor is phosphorylated by Bur1/CDK9 of the P-TEFb complex, facilitating release of RNAPII from its promoter proximal pause site.^26^ At this time, Ser5 is dephosphorylated by the Ssu72/SSU72 phosphatase.^27,28^ During transcription elongation, Bur1/CDK9 and Ctk1/CDK12 phosphorylate the CTD of RNAPII at Ser2 to recruit transcription elongation and splicing factors. CDK9 also phosphorylates Spt5 and the XRN2 torpedo exonuclease to modulate elongation and termination, respectively.^29^ At the 3′ end of genes, phospho-Ser2 recruits termination factors and the 3′ end processing machinery.^30^

Although Ser2 and Ser5 phosphorylation are most prevalent, Tyr1, Thr4, and Ser7 can also be phosphorylated.^31,32^ In yeast, Tyr1 phosphorylation is proposed to prevent premature recruitment of termination factors during elongation.^33^ When RNAPII transcribes the PAS near the end of the 3′ UTR, Tyr1 phosphorylation is removed by the Glc7 phosphatase along with its regulatory subunit, Ref2, to allow phospho-Ser2-mediated recruitment of 3′ end processing and termination factors.^30,33–37^ In mammals, Tyr1 phosphorylation is associated with promoter antisense transcription^38^ and Tyr1 itself has been implicated in transcription termination.^39^ Still, the identity of the Tyr1 kinase remains unknown and Tyr1 phosphorylation is difficult to detect.

Thr4 phosphorylation is involved in transcription termination^40^ and histone pre-mRNA processing^41^ in metazoans, and small nucleolar RNA (snoRNA) termination in yeast.^42^ Ser7 phosphorylation, on the other hand, functions to recruit the Integrator complex to small nuclear RNA (snRNA) genes in metazoans,^43,44^ plays a role in RNAi-mediated heterochromatin formation in fission yeast,^45^ and primes the CTD as a substrate for other CTD kinases and phosphatases.^46^

In summary, phosphorylation of the CTD and transcription factors is thought to strongly influence transcription and pre-mRNA processing. Still, the exact role of each modification is difficult to discern due to the essentiality and pleiotropy of the factors involved. In addition to the post-translational modifications described above, it is likely that there are many additional substrates of the kinases and phosphatases that play a major role in gene expression. To fully understand the function, prevalence, and importance of specific phosphorylations in termination, sophisticated mass spectrometry experiments^47^ alongside genetic perturbations will be required.

## mRNA 3′ End Processing: CPF/CPSF

3′ end processing involves pre-mRNA cleavage and polyadenylation. This releases the mature transcript from transcribing RNAPII so that it can be exported from the nucleus into the cytoplasm for translation. The 3′ end processing machinery includes a large multiprotein complex, called cleavage and polyadenylation factor (CPF) in yeast, or cleavage and polyadenylation specificity factor (CPSF) in humans.^7,8^ CPF/CPSF recognizes the PAS sequence in RNA and also contains the endonuclease activity.^48^ RNA recognition is thought to activate the endonuclease (Ysh1 in yeast; CPSF73 in human) to cleave the nascent transcript 10–30 nucleotides downstream of the PAS, most often after a CA dinucleotide (Figure 1C). The poly(A) polymerase (Pap1 in yeast; PAP in human) is a constitutive subunit of yeast CPF but not human CPSF. Pap1/PAP adds a poly(A) tail onto the new free 3′ end of the cleaved RNA in a template-independent reaction. In addition to CPF/CPSF, accessory factors (CF IA, CF IB in yeast; CstF, CFIIm, RBBP6 in human) play roles in RNA binding and regulation of CPF/CPSF. Overall, at least 14 different proteins within CPF/CPSF and the accessory factors are required for 3′ end processing.^49–51^

In yeast, transcription termination occurs soon after the PAS has been transcribed, thereby preventing transcriptional interference on downstream genes, which are generally in close proximity.^52,53^ In humans, RNAPII can continue hundreds or thousands of nucleotides beyond the PAS before termination takes place. However, PAS recognition, pre-mRNA cleavage, and transcription termination are intimately coupled in all species examined.

### The roles of phosphatases

In addition to an endonuclease and poly(A) polymerase, the multi-subunit CPF complex in yeast also contains two protein phosphatases: Ssu72 and Glc7. Ssu72 dephosphorylates Ser5 and Ser7 of the RNAPII CTD during transcription elongation, while Glc7 dephosphorylates Tyr1 at the 3′ end of genes— possibly in response to the recognition of the PAS sequence in RNA.^7,37^ Because CPF phosphatase activity contributes to transcription termination, CPF couples RNA recognition, 3′ end processing, and transcription termination.

The human orthologs of Glc7 and Ssu72 (protein phosphatase 1 or PP1, and SSU72, respectively) are not constitutive subunits of the CPSF complex. However, PP1 and its regulatory subunit PNUTS, as well as SSU72, associate with a post-cleavage CPSF complex purified from human cell extracts.^54^ Indeed, the role of phosphatases in 3′ end processing and transcription termination is likely to be conserved throughout eukaryotes (see below).

## Transcription Termination Downstream of Protein-Coding Genes

**Figure 2:**
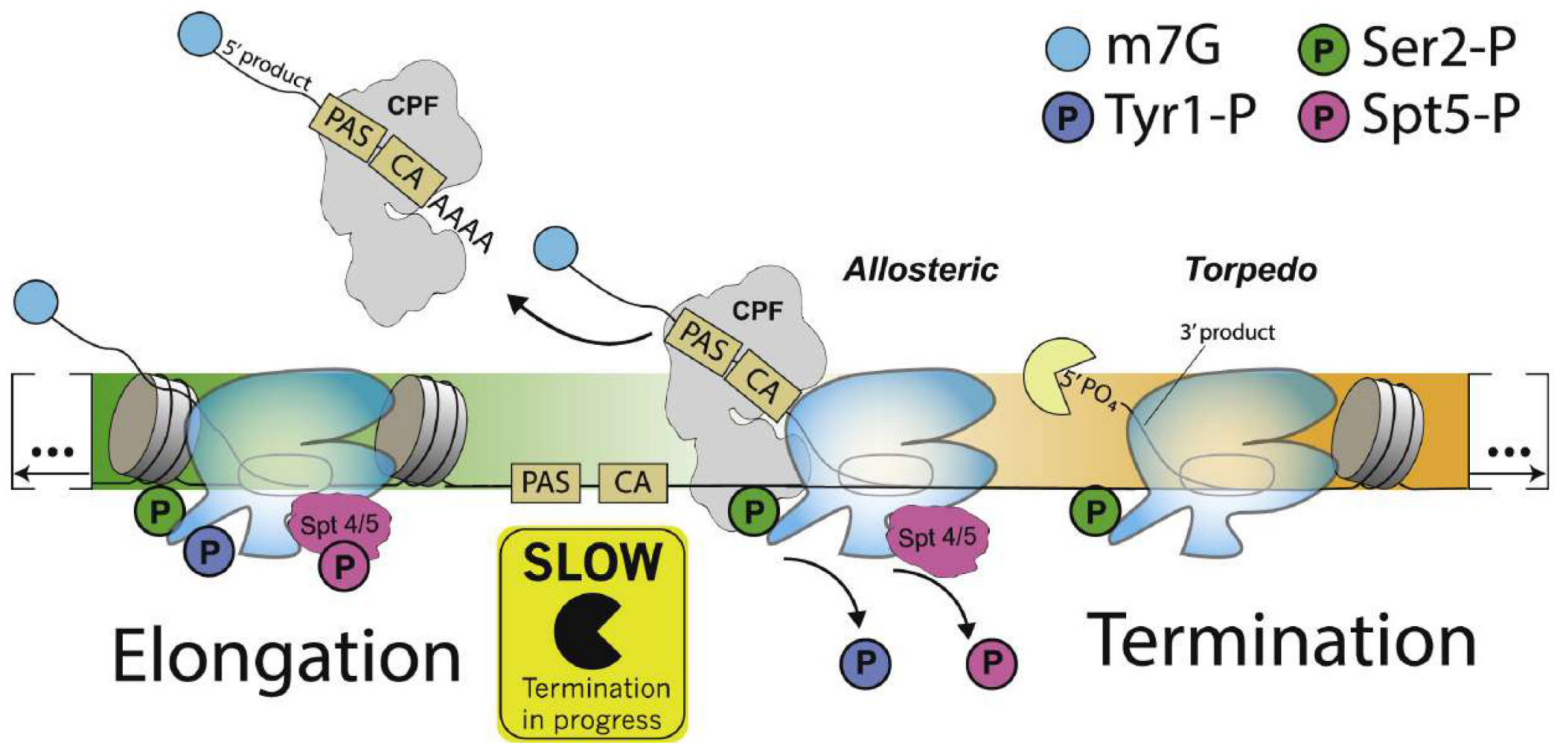
A harmonized model of transcription termination Transcription of the PAS stably recruits CPF/CPSF and associated 3′ processing factors to RNA and transcribing RNAPII. CPF/CPSF binding to the PAS enables activation of its endonuclease activity to cleave the nascent pre-mRNA and release the pre-mRNA from RNAPII. The newly generated 3′ hydroxyl on the 5′ product is the substrate for the poly(A) polymerase Pap1/PAP. The newly generated 5′ phosphate on the 3′ product is the substrate for the torpedo exonuclease, Rat1/XRN2, which is required for termination. CPF/CPSF binding to the PAS may also activate Glc7-Ref2/PP1-PNUTS to dephosphorylate the CTD of RNAPII, which then allows recruitment of termination factors. Thus, recognition of the PAS by CPF/CPSF commits RNAPII to transcription termination through the concerted action of its endonuclease and phosphatase activities. This incorporates aspects of both the allosteric and torpedo models of transcription termination.

Early studies showed that the disruption of pre-mRNA cleavage, for example, by mutations in the PAS or surrounding sequences, results in defects in transcription termination.^55–58^ This dependence on the PAS led to the development of two (non-mutually exclusive) models for transcription termination: the allosteric (or anti-terminator) model and the torpedo model, which are described in detail below^56,57,59^ (Figure 2).

### The allosteric model

In the allosteric model of termination, transcription of the PAS causes a change in RNAPII that promotes termination. The nature of such an allosteric change remains unclear, but it is thought to involve a conformational change in RNAPII that slows elongation and makes it competent for termination and/or the release of a termination inhibitor. Several properties of RNAPII provide support for this model.

Efficient transcription elongation requires phosphorylation of both the Spt5/SPT5 elongation factor and Ser2 of the RNAPII CTD.^60,61^ Transcription of the PAS impacts RNAPII by slowing its speed, and this is thought to promote termination. This likely occurs via the Glc7/PP1 phosphatase and its regulatory subunit, Ref2/PNUTS, which are found in 3′ end processing complexes.^37,54^ Glc7/PP1 dephosphorylates Spt5/SPT5 downstream of the PAS, which slows down RNAPII and enables its termination via the Rat1/XRN2 torpedo (see below).^62–64^ The PP2A phosphatase also antagonizes elongation by dephosphorylating RNAPII and SPT5. However, PP2A is recruited by Integrator near gene promoters and it acts on distinct residues from PP1-PNUTS.^65^

A decrease in the efficiency of RNAPII elongation is paralleled by other changes that promote termination. For example, the transcription of a functional PAS and high levels of phospho-Ser2 at the 3′ ends of genes promote recruitment of the 3′ end processing machinery and termination factors.^30,66,67^ Tyr1 phosphorylation in yeast is thought to prevent premature recruitment of termination factors, thereby acting as an “anti-terminator.”^33^ Selective dephosphorylation of Tyr1 by Glc7/PP1 therefore allows phospho-Ser2 to recruit termination factors, including Pcf11 and Rtt103.^30,36,68–70^ In mammals, phospho-Thr4 is also enriched on RNAPII located beyond the PAS, and this phosphorylation is dependent on CPSF73.^63,71^ This suggests that Thr4 is modified after recruitment of the 3′ end processing machinery and, potentially, as a result of pre-mRNA 3′ cleavage. Phosphorylation patterns of the CTD therefore likely couple pre-mRNA cleavage to transcription termination via the recruitment of termination factors.

At transcription termination, phosphatase activity reverts RNAPII complexes to a hypophosphorylated state, perhaps resembling those at the beginning of transcription in having little elongation competence. It was recently reported that the dephosphorylation of yeast RNAPII results in the formation of an RNAPII dimer that likely cannot bind transcription elongation factors.^16^ Thus, the oligomerization state of RNAPII could also contribute to the slowing of transcription in the allosteric model of termination.

Many pro- and anti-termination factors indirectly influence transcription by creating the conditions for efficient termination—specifically, by modulating RNAPII elongation capacity. For example, the Paf1 complex modulates the elongation capacity of RNAPII to influence elongation, release from promoter-proximal pause, and termination.^72,73^ Pcf11, an accessory factor of the 3′ end processing machinery, has been reported to dissociate elongation complexes from DNA *in vitro*.^74^ Like some other 3′ processing factors, Pcf11 binds both the RNAPII CTD via phospho-Ser2 and RNA. Interestingly, Pcf11 is of low abundance compared with other 3′ end processing factors and may act more selectively than core CPFs. In agreement with this, Pcf11 depletion more strongly affects the transcription of closely spaced genes, where it presumably acts to prevent transcriptional interference.^40^ Notably, Pcf11 can only terminate stalled elongation complexes, which is compatible with the slowing of RNAPII beyond the PAS, as described above.

Stalling or slowing of transcription is a conserved feature of termination. For example, in prokaryotes, factor-independent termination relies on primary and secondary structures within the RNA to arrest RNA polymerase, and eukaryotic RNAPIII can terminate after the transcription of four or more U’s.^75,76^ Sequence elements may also contribute to RNAPII termination in some cases, as U-tracts and other motifs are enriched at the ends of terminated nascent RNA.^77–79^ Such sequences may cause termination on their own but could also improve the process, for example, by facilitating RNAPII stalling.

Finally, the analysis of semi-purified transcription complexes led to a proposal that transcription of the PAS results in conformational changes to RNAPII itself.^80^ This may involve the trigger loop of RNAPII that orients incoming nucleotides. However, it remains unclear whether this is a direct effect on RNAPII conformation and, if so, how this would be mediated. In summary, in the allosteric model, multiple factors, including phosphorylation states and sequence context, are likely to slow RNAPII elongation and promote termination.

### Torpedo model

Terminating without prior 3′ end processing might be dangerous and could release immature (non-processed) pre-mRNA from RNAPII that may ultimately be dysfunctional. Coupling PAS cleavage to termination ensures completion of RNA maturation prior to RNAPII release. This is the basis of the torpedo model for termination, whereby PAS cleavage exposes the RNAPII-associated RNA to a 5′-3′ exonuclease that chases down the polymerase to terminate it.^6,56,59^ The major nuclear 5′-3′ exonuclease is XRN2, and its inactivation or depletion causes termination defects at almost all protein-coding genes.^81–83^ The torpedo exonuclease in budding yeast is Rat1, and it functions in complex with the Rai1 and Rtt103 proteins.^34^

In the torpedo model, Rat1/XRN2 co-transcriptionally degrades the 3′ cleavage product continuously until it reaches RNAPII. In agreement with this, XRN2 initiates degradation at the cleavage site and XRN2 depletion stabilizes RNA downstream of the PAS.^81,84^ Moreover, placing XRN2-resistant structures downstream of the PAS impairs termination, suggesting that RNA directly connects XRN2 activity and RNA polymerase.^63^ As discussed above, transcription of the PAS slows RNAPII—this likely facilitates termination by XRN2. Indeed, there is a correlation between polymerase speed and the site of termination. RNAPII mutants that are slowed tend to terminate at upstream sites, whereas faster RNAPII variants terminate further downstream.^82^

XRN2-dependent termination can also occur in a PAS-independent manner, for example, in response to RNaseP activity at NEAT1 and MALAT1 non-coding genes.^81,82^ Moreover, antisense oligonucleotides (ASOs) can direct RNaseH1-dependent RNA cleavage and subsequent XRN2-dependent transcription termination.^63,85,86^ This is not dependent on a PAS, again suggesting that pre-mRNA cleavage, but not the 3′ end processing machinery itself, is required for XRN2-dependent termination. It is possible that ASOs direct RNA cleavage more rapidly than a PAS and negate the need to slow RNAPII. Alternatively, RNA cleavage itself could trigger RNAPII slowing. Importantly, not every co-transcriptionally formed 5′ phosphate promotes XRN2-dependent termination: XRN2 elimination does not affect termination at snRNA and histone genes, although 3′ end processing at both transcript classes involves an endonuclease^81^ (see below). Interestingly, inactive XRN2 impairs the degradation of histone 3′ end cleavage products and mildly affects subsequent termination, perhaps by loading onto the 5′ end of the cleaved RNA and blocking access to a redundant exonuclease.^82,87^ Finally, XRN2 is implicated in the promoter-proximal termination of RNAPII and, although the nature of its entry site is not defined, possibilities include the 5′ end of incompletely capped RNA or the new end generated by Integrator cleavage.^87^

A major question is how XRN2 causes RNAPII to release the template. The torpedo model originally implied that exonuclease activity would forcefully dislodge RNAPII, but it remains unclear whether this is the case or how it may occur. Because inactive mutants of Rat1/XRN2 cannot support efficient termination,^34,81,82^ it seems likely that their activity forms a key part of this process. Analogous mechanisms are present in bacteria, some of which utilize the exonuclease activity of RNaseJ1 for termination or the helicase activity of Rho.^88,89^ Structures are available for the latter, which, together with biochemical studies,^88,90^ suggest Rho activity pulls on RNA or causes forward translocation of the polymerase. Even so, RNA degradation may not be sufficient for termination because Rat1 activity cannot dislodge *E. coli* RNA polymerase from DNA *in vitro* and nuclear Xrn1 cannot promote termination in yeast cells lacking Rat1.^91,92^ This suggests that Rat1 (and XRN2) may form specific contacts that are important for terminating eukaryotic RNAPII. Additional factors discussed above may also improve the process. Interestingly, structures of Rho-dependent termination demonstrate that NusG bridges Rho with RNA polymerase.^90^ NusG is the homolog of Spt5, which plays a key role in RNAPII elongation competence, as outlined above.

### A unified model of transcription termination

The allosteric and torpedo models are compatible with each other. Transcription of the PAS allows the 3′ end processing machinery to assemble on RNA. This would activate the 3′ endonuclease and protein phosphatase activities, resulting in cleavage of the pre-mRNA and changes in the phosphorylation state of the CTD (Figure 2). This would in turn allow the torpedo exonuclease to access the downstream RNA and would impair efficient RNAPII elongation. Together, these multiple signals coordinate timely 3′ end processing and transcription termination.

### Preventing premature termination: Telescripting

During transcription elongation, premature transcription termination and the use of intragenic PAS sequences is inhibited. Specifically, splicing factors, U1 snRNA, SCAF4, and SCAF8, as well as the kinase CDK12, inhibit the action of the 3′ end processing machinery at intronic PAS sites.^93–96^ As such, interfering with the function of these factors causes premature mRNA 3′ end processing and transcription termination in human cells.^95,97^ This is best-characterized in the case of U1 snRNA, which masks intronic PAS sites in a process called telescripting.^95^ Telescripting is independent of the role of U1 in splicing and is enabled by the fact that U1 snRNA levels exceed those of other spliceosomal snRNAs, presumably allowing it to occupy additional sites within introns and suppress nearby PASs. Consistently, core components of U1 snRNP (U1A, U1C, and U1-70k) crosslink together with cleavage factors at intronic PAS sequences that are activated when U1 snRNA telescripting is inhibited.^98^

A critical issue is how telescripting is alleviated to allow correct PAS usage at the ends of genes. In internal exons, there is normally another 5′ splice site (that can recruit U1 snRNP) downstream of a given 3′ splice site, which may promote telescripting in the subsequent intron. However, at the end of the gene, the terminal 3′ splice site is instead followed by a PAS, which may favor 3′ end processing over further telescripting.^99^ This arrangement underpins the elongation to termination transition by allowing recognition and processing of the correct PAS. Intriguingly, both the 3′ end processing machinery and U1 snRNP interact directly with RNAPII near the RNA exit channel.^16,18^ A physical interplay between these complexes at this site could influence telescripting.

## Other Termination Pathways

In addition to the poly(A)-dependent mechanism, transcription can terminate via a variety of alternative pathways. Human histone mRNAs are processed by a machinery that contains U7 snRNA and also shares some subunits with CPSF, including the CPSF73 endonuclease.^100^ As mentioned above, XRN2 does not play a major role in termination at histone genes in humans. Thus, allosteric termination may be more prominent on histone genes, although it is worth noting that CPSF73 can act as an exonuclease that may substitute for XRN2.^101^ While CPSF binds upstream of the PAS cleavage site at protein-coding genes, U7 snRNA tethers the histone cleavage complex downstream of the cleavage site, which could better-position CPSF73 to initiate degradation of the downstream RNAPII-associated product and potentially serve as an alternative torpedo exonuclease.

Primary snRNA transcripts are also processed endonucleolytically via the Integrator complex, within which INTS11 provides cleavage activity.^102^ Despite this, termination at snRNA genes is insensitive to XRN2 and so could employ an allosteric mechanism or an alternative exonuclease. INTS11 is highly similar to CPSF73, although it is unknown whether it has exonuclease activity. Integrator also mediates premature transcription termination at protein-coding genes that controls their ultimate output.^103–105^ Transcription termination by the Integrator complex is covered in detail in a parallel review by Adelman and colleagues.^5^

In fission yeast, termination of snoRNA genes is dependent on the CPF endonuclease, the torpedo exonuclease, and, in some circumstances, the exosome, which is proposed to act as a “reverse torpedo” in a 3′-5′ direction.^106^ However, in budding yeast, termination and 3′ end processing of short non-coding RNAs, including snRNAs and snoRNAs, occurs via a PAS-independent mechanism involving the Nrd1-Nab3-Sen1 (NNS) complex.^107,108^ Recruitment of Nrd1-Nab3 to elongating RNAPII is mediated through the recognition of phospho-Ser5 on the CTD of RNAPII.^109,110^ Sequence elements on nascent pre-snoRNAs also contribute to Nrd1-Nab3 recruitment.^111^ Sen1, a 5′-3′ ATP-dependent superfamily 1 helicase, is recruited through multiple contacts with RNAPII and the Nrd1-Nab3 heterodimer.^112,113^ Sen1 likely promotes termination of short non-coding RNA genes similar to Rho and XRN2, whereby the activity of a processive enzyme tracks down RNAPII to disengage it from chromatin.^114^ Sen1-mediated termination is sensitive to RNAPII elongation rates, in agreement with a requirement for slowed transcription for efficient termination.^115^

Interestingly, in budding yeast, six subunits of CPF (the phosphatase module) associate with the Syc1 protein to form the associated with Pta1 complex (APT), which contributes to transcription of non-coding RNAs.^116^ The APT complex contains both of the CPF phosphatases, and these are required for transcription termination on non-coding RNAs, likely by dephosphorylating Sen1 and the RNAPII CTD.^117,118^ Similarly, phosphatases within the Integrator complex play an important role in termination.^119^ Other components of the cleavage and polyadenylation machinery also play a role in poly(A)-independent termination. For example, Pcf11 contributes to termination on snoRNAs, possibly in concert with Rtt103, which binds phospho-Ser2 and phospho-Thr4.^34,42,120–122^ Thus, it appears that although cleavage and polyadenylation activities are only required on a subset of RNAPII-transcribed RNAs, the phosphatase activities as well as the accessory factors (CF IA) are more generally required for the termination of RNAPII transcripts.

## Regulation of Transcription Termination

**Figure 3:**
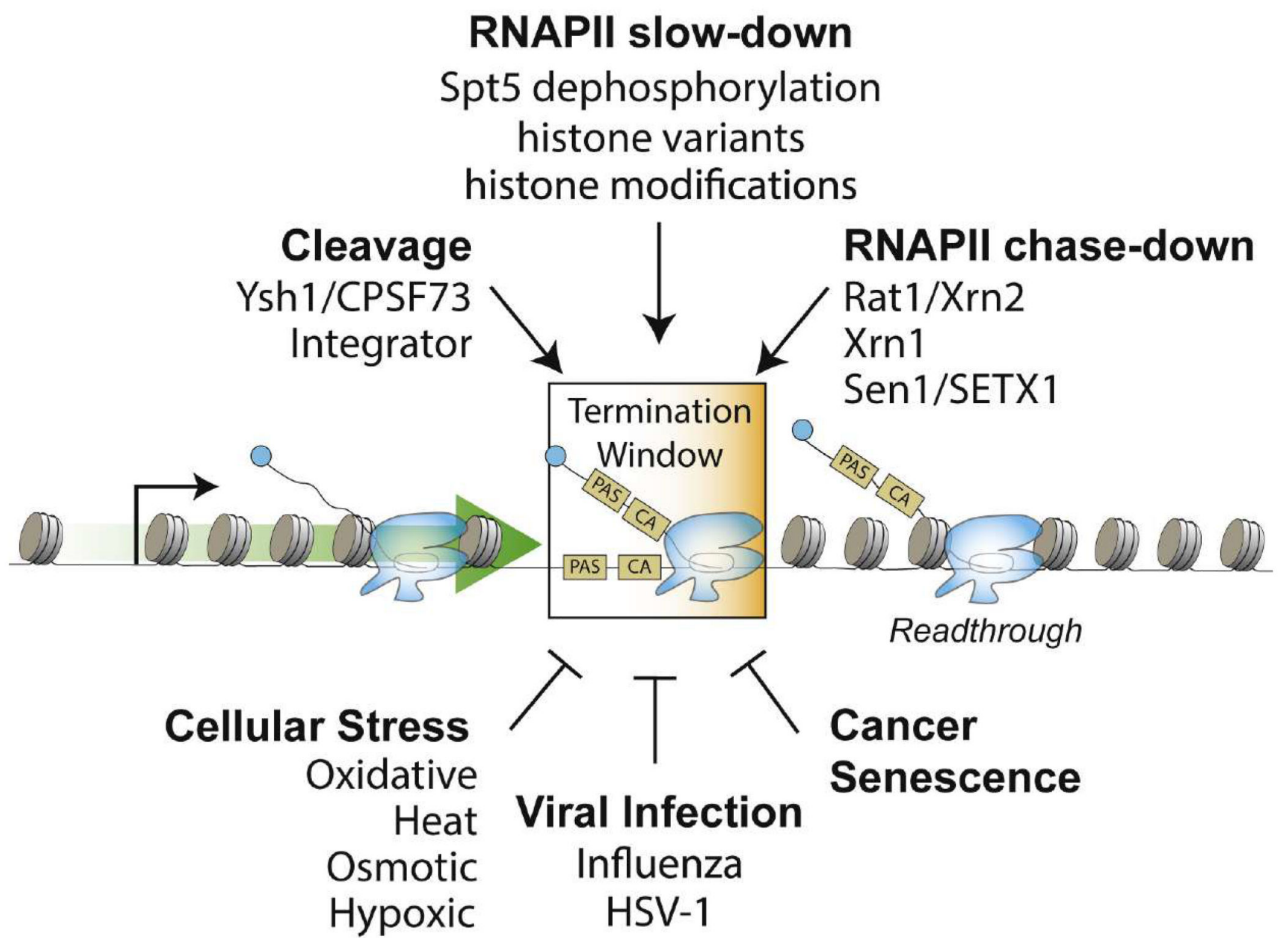
Regulation of termination by multiple mechanisms and cellular signals Summary of the factors, mechanisms, and cellular conditions that promote (top) and impair (bottom) transcription termination by RNAPII.

Alteration of transcription termination in response to cellular cues likely regulates gene expression (Figure 3). Mounting evidence suggests that transcription termination is impaired during environmental stress, including heat, hypoxia, osmotic shock, and oxidative stress. Termination is also compromised during cellular senescence, viral infection, and certain cancers. In most cases, altered termination occurs on a common class of genes with shared features, including a weak PAS sequence, open chromatin, and proximity to other genes. This would suggest that some genes are predisposed to altered termination in response to external stimuli.

### Environmental stress

During heat, osmotic, or oxidative stress, transcription termination is impaired and gives rise to “downstream of gene” transcripts (DoGs).^123^ DoGs are continuous with their associated upstream mRNA transcripts and do not represent *de novo* transcription initiation events. They are depleted of strong PAS sequences, are retained in the nucleus, and are not translated.^123,124^ Thus, DoGs represent *bona fide* readthrough transcripts. DoGs do not appear to be unstable and instead show half-lives similar to the median mRNA half-life (1 h). They have been detected in both poly(A)− and poly(A)+ mRNA fractions of cells undergoing osmotic shock. The production of DoGs is linked to reduced Integrator function.^125^

It has been proposed that DoGs carry out important physiological functions during cellular stress responses. When cells are removed from stress conditions, DoGs show stress-specific recovery rates as they return to basal pre-stress levels.^126^ This has been interpreted as DoGs being under modes of stress-specific regulation, and potentially playing a role in recovery from stress conditions. Moreover, because some DoGs remain tethered to chromatin, one hypothesis is that DoGs function globally in maintaining nuclear integrity during stress.^123^ Alternatively, transcription readthrough itself may regulate downstream genes by transcription interference.^127^ Interestingly, DoGs tend to be more prevalent if there is a downstream gene in close proximity (irrespective of orientation).^126^ Systematic studies that rescue the termination defects that give rise to DoGs will be critical in assessing their function and understanding how they are generated.

### Viral infection

During herpes simplex virus 1 (HSV-1) infection, transcription termination is impaired.^128^ This is largely mediated by the viral RNA-binding protein ICP27, which interacts with CPSF and disrupts the assembly of a functional complex.^129^ Surprisingly, ICP27 can also bind GC-rich sequences upstream of the PAS, which paradoxically results in the recruitment of CPSF and premature transcription termination.^130,131^ The molecular details of the dual function of ICP27 remain unclear. Further structural and biochemical studies using the recently reported reconstituted CPSF complex^49,51^ could help explain how ICP27 can function to both aid and antagonize CPSF function and transcription termination.

During influenza A infection, host gene expression is disrupted by defects in transcription termination and the depletion of transcribing RNAPII near the 5′ ends of genes.^132^ The termination defect is mediated, at least in part, by the non-structural viral protein, NS1A, which directly interacts with the CPSF30 subunit of CPSF.^66,133,134^ Exactly how NS1A inhibits CPSF activity is not understood. It is clear, however, that NS1A interferes with the binding of CPSF to its RNA substrate.^134^ Biochemical and structural studies show that two copies of NS1A interact with two copies of the RNA-binding zinc fingers of CPSF30. This interaction may disrupt CPSF30 assembly into CPSF or it may induce the dimerization of CPSF to favor an inactive form of the complex. In addition, the influenza A viral polymerase stabilizes the NS1A-CPSF30 interaction^135^ and interacts with the Ser5 phosphorylated form of RNAPII near the 5′ end of genes, where it can sequester CPSF away from the site of 3′ end processing.^136,137^

Divergent influenza strains with orthologs of NS1A that are not predicted to interact with CPSF30 also impair transcription termination, leading to transcription readthrough reminiscent of DoG production in oxidative stress.^132^ Thus, cellular stress may generally lead to transcription termination defects that primarily affect transcripts lacking strong PAS sites.^124^ Further studies are required to understand whether these defects in transcription termination are specifically induced or whether they are the general consequence of cellular stress.

### Cancer and senescence

Transcription termination defects have also been observed in a number of cancers, and this can correlate with poor prognosis.^138^ In clear cell renal cell carcinoma, readthrough correlates with mutations in the *SETD2* gene, which methylates histone H3 lysine 36 (H3K36me3), suggesting that there is a link between transcription termination and chromatin marks of active transcription.^138^ Readthrough transcription can lead to novel chimeric fusion transcripts or circular isoforms,^139^ which arise from *trans-splicing* and back-splicing events between adjacent co-directional genes.^140^ It is likely that chimeric fusion transcripts are a general feature of transcription termination defects,^141^ and may even produce functionally novel proteins.

In cells undergoing oncogene-induced senescence, termination defects between a small subset of convergent genes gives rise to senescence-triggered antisense readthrough (START) RNAs.^142^ START transcripts appear to repress the expression of adjacent convergent genes and promote the senescent gene expression program and permanent cell proliferation arrest. START RNAs are stable transcripts and arise from increased readthrough transcription due to a defect in RNAPII slow down within the termination window. Although it remains unclear why elongating RNAPII fails to slow down, the H2A histone variant H2A.Z appears to play a role in repressing START RNAs.

### Regulation of telescripting

Telescripting may also be regulated by external inputs. For example, heat shock causes premature transcription termination as well as termination defects at the 3′ ends of genes.^143^ Many of the prematurely terminated genes contain multiple introns and strong intronic PAS sequences, consistent with impaired telescripting.^96^ Surprisingly, heat shock also increases the transcription rate of elongating RNAPII.^143^ A faster moving RNAPII could miss the termination window near the 3′ ends of genes, giving way to runaway transcription elongation complexes.^82,94^ This would be reminiscent of the way weak splice sites can be ignored when elongation is fast.^144^

A tantalizing idea that emerges from these studies is that cellular stressors, disease states, and viral infections may cause early recognition of the PAS and premature transcription termination through independent mechanisms. For example, cap snatching of the 5′ end of the U1 snRNA^145^ would reduce U1 snRNA transcript levels, impair telescripting, and increase intronic cleavage and polyadenylation, followed by premature transcription termination at protein-coding genes. During HSV-1 infection, on the other hand, ICP27-mediated recruitment of CPSF to PAS sites near GC-rich regions would cause premature termination independently of telescripting.^131^ A stress-induced shift in U1-snRNP abundance could also tip a delicate balance between telescripting and transcription termination.^143^ Finally, the heat-responsive master regulator heat-shock factor 1 (HSF1), directly interacts with the 3′ end processing machinery in a heat-shock-dependent manner,^146^ and may recruit CPSF to heat-responsive genes, which are typically shorter, have fewer introns, and are not subject to telescripting.^96^

## Outlook

Termination has been the least-studied phase of the transcription cycle, perhaps because it happens after RNAPII is perceived to have already done its important jobs. As this review highlights, we are now beginning to understand its mechanisms, as well as appreciate that termination plays important roles in gene expression beyond evicting RNAPII from chromatin. Still, there are many interesting questions remaining. Although recent data unify the allosteric and torpedo models, we still do not know how RNAPII is dislodged from its template. Cryo-EM offers the most exciting avenue to pursue this, having already illuminated critical RNA processing mechanisms in detail, including prokaryotic transcription termination.^147,148^ Studies of the kinase and phosphatase components of the transcription cycle have given critical insight into the importance of RNAPII elongation control. It seems certain that the substrate repertoire of these is much broader than currently appreciated and identification of new targets will further improve our understanding of termination. Similarly, SPT5 is central to RNAPII elongation, but we do not understand how its phosphorylation influences critical protein-protein interactions. Lastly, is XRN2 the only “torpedo” or does CPSF73 (and even INTS11) also have torpedo exonuclease activity in certain contexts?

Once RNAPII escapes the promoter, complete elongation across a gene was once thought inevitable. However, premature termination is commonplace and, on many genes, it may seal the fate of the majority of elongating RNAPIIs.^119^ That this process is sensitive to external inputs such as heat shock suggests an underappreciated regulatory capacity that will be interesting to elucidate in the future. CPSF is already known to be targeted by influenza and HSV, as well as being targeted in cancer, but there may be more ways of conditionally influencing transcription termination. It is likely that altered 3′ end processing and transcription termination are as highly regulated and as prevalent in disease as changes in splicing. Understanding how transcription terminates has led to a transformation in our understanding of the transcription cycle in general. Far from being at the end, we may be just beginning to understand its many roles in gene regulation.
